# Development of a predictive model for identifying women vulnerable to HIV in Chicago

**DOI:** 10.1186/s12905-023-02460-7

**Published:** 2023-06-16

**Authors:** Eleanor E. Friedman, Shivanjali Shankaran, Samantha A. Devlin, Ekta B. Kishen, Joseph A. Mason, Beverly E. Sha, Jessica P. Ridgway

**Affiliations:** 1 Department of Medicine, University of Chicago, 5841 S. Maryland Ave, MC 5065, Chicago, IL 60637 USA; 2 Rush University Medical Center, Chicago, IL USA

**Keywords:** HIV, HIV vulnerability, Prediction model, Electronic Medical Record (EMR), PrEP, Risk factors

## Abstract

**Introduction:**

Researchers in the United States have created several models to predict persons most at risk for HIV. Many of these predictive models use data from all persons newly diagnosed with HIV, the majority of whom are men, and specifically men who have sex with men (MSM). Consequently, risk factors identified by these models are biased toward features that apply only to men or capture sexual behaviours of MSM. We sought to create a predictive model for women using cohort data from two major hospitals in Chicago with large opt-out HIV screening programs.

**Methods:**

We matched 48 newly diagnosed women to 192 HIV-negative women based on number of previous encounters at University of Chicago or Rush University hospitals. We examined data for each woman for the two years prior to either their HIV diagnosis or their last encounter. We assessed risk factors including demographic characteristics and clinical diagnoses taken from patient electronic medical records (EMR) using odds ratios and 95% confidence intervals. We created a multivariable logistic regression model and measured predictive power with the area under the curve (AUC). In the multivariable model, age group, race, and ethnicity were included a priori due to increased risk for HIV among specific demographic groups.

**Results:**

The following clinical diagnoses were significant at the bivariate level and were included in the model: pregnancy (OR 1.96 (1.00, 3.84)), hepatitis C (OR 5.73 (1.24, 26.51)), substance use (OR 3.12 (1.12, 8.65)) and sexually transmitted infections (STIs) chlamydia, gonorrhoea, or syphilis. We also a priori included demographic factors that are associated with HIV. Our final model had an AUC of 0.74 and included healthcare site, age group, race, ethnicity, pregnancy, hepatitis C, substance use, and STI diagnosis.

**Conclusions:**

Our predictive model showed acceptable discrimination between those who were and were not newly diagnosed with HIV. We identified risk factors such as recent pregnancy, recent hepatitis C diagnosis, and substance use in addition to the traditionally used recent STI diagnosis that can be incorporated by health systems to detect women who are vulnerable to HIV and would benefit from preexposure prophylaxis (PrEP).

**Supplementary Information:**

The online version contains supplementary material available at 10.1186/s12905-023-02460-7.

## Introduction

The HIV epidemic remains a major public health challenge in the United States, with 36,801 people newly diagnosed with HIV in 2019. The majority of these new cases occurred among those born male who identify as male (79%) and via male-to-male sexual contact (66%) [1]. Despite representing lower absolute numbers of new HIV cases, women constituted 19% of incident HIV cases, with the majority of these cases attributed to heterosexual contact [2]. Women, particularly African American/Black women, remain at risk for HIV due to complicated interactions between multiple levels of HIV risk, including community and structural level factors, such as widespread poverty and constrained sexual networks [3, 4]. These complex risk factors among women make it difficult to identify those who are most vulnerable to HIV and in need of pre-exposure prophylaxis (PrEP), a group of medications that are highly effective at preventing HIV [5]. Consequently, a large number of the PrEP-eligible indications for women are based not on their individual behavior, but rather on the behavior of their partners, behavior that may not be known by these women [6, 7].

The difficulty in readily identifying HIV risk factors for women also has implications for technology designed to aid in HIV prevention efforts. In the last decade, multiple models have been built to identify persons at risk for HIV, persons who have not been diagnosed with HIV, or persons who are eligible for PrEP. These models have used data from a variety of countries and have included a wide range of cultural and region-specific factors that influence both HIV care and HIV prevention efforts to identify those most in need of intervention [8–13]. One subset of these models has examined using data from electronic medical records (EMR) to identify persons eligible for PrEP. Models from the United States that either used HIV diagnosis as their outcome or were later validated among people who became HIV positive have shown poor predictive performance among women [8, 14−17]. A consequence of using all new HIV diagnoses or using persons eligible for PrEP is that the models are composed mostly of men, and thus the risk factors identified by these models are also biased toward men. This can be seen in the inclusion of variables such as being male, MSM sexual behavior, and medications for erectile dysfunction [8, 16]–[17]. This finding is particularly unfortunate and paradoxical given that women are more likely than men to have a history of preventative healthcare use, [18] which would result in the creation of EMRs that could be used in HIV risk models.

Obstetrics and Gynecology (OBGYN) and emergency department (ED) settings may be of particular importance in identifying women at high risk for HIV. A recent study found that 82% of women newly diagnosed with HIV had prior healthcare encounters that represented missed opportunities for PrEP initiation, 84% of which occurred in the ED [19]. Most women utilize family or internal medicine doctors for primary care, but OBGYNs are more often used as a type of primary provider among people who are uninsured versus people with insurance (12% vs. 7%) and Black women versus white women (12% vs. 6%) [20]. Additionally, between 2007 and 2010, OBGYN appointments represented 44% of all preventative care visits among women, focusing primarily on reproductive health-related services. Accordingly, OBGYN is a setting well suited to deliver PrEP counseling [21, 22].

The lack of predictive ability for women from previous models that incorporate both men and women may be due to sex-specific HIV risks, as well as the relatively smaller number of women who became HIV positive. The creation of an HIV prevention model specifically among vulnerable women may discover risk factors and result in increased ability to identify women. To determine significant HIV risk factors specifically among women, we established a cohort of women to create an EMR-based risk assessment model for women who underwent routine HIV testing.

## Methods

Data were drawn from two large opt-out HIV testing programs embedded within hospitals in Chicago, Illinois [23]. Inclusion criteria for this study included: (1) female sex (i.e., a person whose legal sex was female) (2) underwent testing for HIV between 1/1/2014 to 3/31/2020, and (3) had either an outpatient OBGYN or ED visit from 1/1/2014 to 3/31/2020 at Rush University Medical Center (RUMC) or University of Chicago Medicine (UCM). This study was approved by the Institutional Review Board of UCM. The Institutional Review Board of UCM served as the IRB of record for RUMC. A waiver of consent was sought and given to obtain retrospective EMR data from women tested for HIV at these institutions. We collected information regarding sociodemographic characteristics, risk behaviors, infectious diseases and other diseases, and HIV testing from patients’ EMRs. We also included data from laboratory results, medical encounters, and social history forms. Both institutions use the same EMR system (Epic), making data extraction and combination straightforward. Only variables that were consistent (i.e., in terms of both presence/absence and how they were measured) across both systems were combined and included in analysis.

To examine differences in sociodemographic, behavioral, and medical history between those who were newly diagnosed with HIV and those who were not, we selected a subset of women who tested negative for HIV using propensity score matching based on site of care and number of prior encounters in the healthcare system. Four HIV-negative patients were chosen for each woman newly diagnosed with HIV using optimal fixed ratio matching with a caliper difference of 0.25 of the logit of the propensity score. Propensity score matching was done to minimize differences in healthcare utilization, as well as amount of information available in the EMR between those newly diagnosed with HIV and those without HIV. To ensure that we were using only women newly diagnosed with HIV, we confirmed HIV status (i.e., newly diagnosed vs. an existing case) with the Chicago Department of Public Health (CDPH) [24, 25].

After the analytic sample was chosen, we examined the following variables: sociodemographic variables, including race, ethnicity, age, education level, and zip code; behavioral information, including self-reported gender of sexual partners, condom use, and being sexually active; infectious disease information, including diagnosis with hepatitis C and diagnoses of chlamydia, gonorrhea, or syphilis (hereafter referred to as sexually transmitted infections (STIs)), and HIV testing. Other health diagnoses that we examined included mental health disorders (e.g., mood disorders, personality disorders, psychosis, and anxiety), substance use (e.g., sedatives, stimulants, opiates, and cannabis), alcohol use, and pregnancy. All diagnoses were examined using ICD-9/10 CM codes, with the absence of particular diagnosis codes within the EMR considered a lack of those associated diseases (Additional File Table 1). Ultimately for all variables, we limited data to a two-year retrospective period prior to their last medical encounter (for HIV negative individuals) or two years prior to HIV diagnosis date. However, we did explore multiple time intervals, particularly for pregnancy, which has a progression that can be difficult to identify using some ICD 9 pregnancy diagnosis codes.

Differences in characteristics between women newly diagnosed with HIV and women without HIV were described using chi-square tests or Fisher’s exact test as necessary. Bivariate and multivariate logistic regression models were created to examine associations, reporting odds ratios (or adjusted odds ratios) and 95% confidence intervals (OR, aOR, 95%CI). For all models, complete case analysis was used. Several variables were entered into the model a priori based on their importance in contributing to disparities between women newly diagnosed with HIV and women without HIV. These variables included age category, race, and ethnicity. We also included variables that were used in the matching process (healthcare site and maximum number of encounters) and any variables that were found to be statistically significant (p-value ≤ 0.05) at the bivariate level. The top performing final logistic regression model was chosen based on both parsimony as well as having a high area under the curve (AUC) value and was compared to simpler models using the receiver operating curve (ROC) contrast test. All methods were carried out in accordance with relevant guidelines and regulations. All analyses were conducted using SAS version 9.4 (SAS Institute INC. Cary, North Carolina). This manuscript meets the “strengthening the reporting of observational studies in epidemiology” (STROBE) guidelines.

## Results

Overall, we identified 55,736 women who underwent HIV testing between 1/1/2014 and 3/31/2020. This included 27,965 women at RUMC and 27,771 women at UCM. Out of this population, we identified 48 women newly diagnosed with HIV, and using propensity score matching, matched 192 women without HIV to these cases by healthcare site and number of prior medical encounters. Propensity score matching reduced the variability in the overall number of previous medical encounters ever from a median of 49 (range 1-5982) to a median of 47.5 (range 5-602).

After matching by site and number of encounters, the analytic sample contained 240 women. These women were mostly African American/Black (67.9%), non-Hispanic/Latina (85.4%), and from either the West (34.2%) or the South (30.8%) sides of Chicago (Table 1). Most women did not have information on their educational attainment (65.0%); however, the most commonly reported level was some college education (12.5%). In terms of medical diagnoses, pregnancy (26.3%) and mental health disorders (24.6%) were seen in about a quarter of the study population; STI diagnoses (0.8%) and hepatitis C diagnosis (2.9%) were much less common. Among women diagnosed with hepatitis C, 71.4% also had diagnosis code indicating substance abuse.

**Table 1:** Characteristics of propensity score matched sample, comparing those who are HIV negative and those who are HIV positive (N = 240)

Variable	Total population	HIV- (N = 192)	HIV+ (N = 48)	Chi square p-value
**Race**				
White African American Other/Unknown	46 (19.7%) 163 (67.9%) 31 (12.9%)	43 (22.4%) 121 (63.0%) 28 (14.6%)	3 (6.3%) 42 (87.5%) 3 (6.3%)	0.005
**Ethnicity**				
Non-Hispanic/Latino Hispanic/Latino	205 (85.4%) 35 (14.6%)	160 (83.3%) 32 (16.7%)	45 (93.8%) 3 (6.3%)	0.07*
**Age**				
18–26 27–35 36–47 48 and older	63 (26.3%) 63 (26.3%) 55 (22.9%) 59 (24.6%)	46 (24.0%) 53 (27.6%) 42 (21.9%) 51 (26.6%)	17 (35.4%) 10 (20.8%) 13 (27.1%) 8 (16.7%)	0.21
**Education**				
Null Some high school Complete High School Some college College degree or higher	156 (65.0%) 8 (3.3%) 24 (10.0%) 30 (12.5%) 22 (9.2%)	126 (65.6%) 5 (2.6%) 16 (8.3%) 26 (13.5%) 19 (9.9%)	30 (62.5%) 3 (6.3%) 8 (16.7%) 4 (8.3%) 3 (6.3%)	0.22*
**Side of city**				
Not Chicago/Unknown Central/Northside Southside Westside	62 (25.85) 22 (9.2%) 74 (30.8%) 82 (34.2%)	51 (26.6%) 18 (9.4%) 60 (31.3%) 63 (32.8%)	11 (22.9%) 4 (8.3%) 14 (29.2%) 19 (39.6%)	0.90*
**Healthcare site**				
Rush UCM	145 (60.4%) 95 (39.6%)	116 (60.4%) 76 (39.6%)	29 (60.4%) 19 (39.6%)	0.99
**Number of encounters**				
(Median, IQR)	47.5 (15–175)	47.5 (15–175)	47.5 (15–175)	0.99
**Hepatitis C**				
No Yes	233 (97.1%) 7 (2.9%)	189 (98.4%) 3 (1.6%)	44 (91.7%) 4 (8.3%)	0.03*
**Substance use**				
No Yes	223 (92.9%) 17 (7.9%)	182 (94.8%) 10 (5.2%)	41 (85.4%) 7 (14.6%)	0.05
**Alcohol use**				
No Yes	236 (98.3%) 4 (1.7%)	190 (99.0%) 2 (1.0%)	46 (95.8%) 2 (4.2%)	0.18*
**Mental health disorder**				
No Yes	181 (75.4%) 59 (24.6%)	145 (75.5%) 47 (24.5%)	36 (75.0%) 12 (25.0%)	0.94
**STIs**				
No Yes	238 (9.2%) 2 (0.8%)	192 (100.0%) 0 (0.0%)	46 (95.8%) 2 (4.2%)	0.04*
**Pregnancy**				
No Yes	177 (73.8%) 63 (26.3%)	147 (76.6%) 45 (23.4%)	30 (62.5%) 18 (37.5%)	0.05
**Male partner**				
Not noted/no Yes	152 (63.3%) 88 (36.7%)	121 (63.0%) 71 (37.0%)	31 (64.6%) 17 (35.4%)	0.84
**Condom use**				
Not noted/no Yes	224 (93.3%) 16 (6.7%)	181 (94.3%) 11 (5.7%)	43 (89.6%) 5 (10.4%)	0.24
**Sexually active data present**				
No Yes	109 (45.4%) 131 (54.6%)	88 (45.8%) 104 (54.2%)	21 (43.8%) 27 (56.3%)	0.80

*Fisher’s exact test was used due to small sample size.

When examining alternate time intervals before HIV diagnosis, we discovered that similar numbers of women had a pregnancy associated diagnosis code zero to six months prior to their HIV diagnosis (17/18, 94.4%) as had a pregnancy diagnosis code zero to 12 months or zero to 24 months before their HIV diagnosis (18/18, 100.0%).

In our sample, African American/Black women had nearly five times the odds of being newly diagnosed with HIV compared to white women (OR 4.98 95%CI (1.47, 16.90)). Women who were of Hispanic or Latina heritage had lower odds of being newly diagnosed with HIV versus those who were not Hispanic/Latina (OR 0.33 95%CI (0.10, 1.14)), although this result was not statically significant. Pregnant women had nearly twice the odds of acquiring HIV than those who were not pregnant (OR 1.96 95%CI (1.00, 3.84)). Similarly, those with hepatitis C had five times the odds of being diagnosed with HIV than those without hepatitis C (OR 5.73 95%CI (1.24, 26.51)). Having been diagnosed with a bacterial STI was significant in Fisher’s exact testing (Fisher’s exact p-value 0.04). Women who had diagnoses of substance use had three times the odds of being newly diagnosed with HIV compared to women who did not have substance use in their medical history (OR 3.12 95%CI (1.12, 8.65)) (Table 2).

**Table 2:** Unadjusted and adjusted odds ratios and 95% confidence intervals for a priori variables, or those that were significant in chi-square or Fisher’s exact testing

Variable	Odds ratio OR (95% CI)	Wald chi-square p - value	Adjusted odds ratio aOR (95% CI)	Wald chi-square p - value
Healthcare site				
RUMC UCM	REF 1.00 (0.52, 1.91)	0.99	REF 1.20 (0.52, 2.76)	0.67
Number of encounters	1.00 (1.00, 1.00)	0.99	1.00 (1.00, 1.00)	0.59
Race				
White African American Other/Unknown	REF 4.98 (1.47, 16.90) 1.54 (0.29, 8.16)	0.003 0.59	REF 4.87 (1.21, 19.53) 1.39 (0.23, 8.35)	0.03 0.57
Ethnicity				
Non-Hispanic/Latino Hispanic/Latino	REF 0.33 (0.10, 1.14)	0.08	REF 0.89 (0.16, 4.97)	0.89
Age				
18–26 27–35 36–47 ≥ 48	REF 0.51 (0.21, 1.23) 0.84 (0.36, 1.93) 0.42 (0.17, 1.08)	0.42 0.37 0.17	REF 0.62 (0.24, 1.65) 0.88 (0.34, 2.31) 0.37 (0.12, 1.17)	0.83 0.37 0.12
Substance use				
No Yes	REF 3.12 (1.12, 8.65)	0.03	REF 1.94 (0.49, 7.64)	0.34
Hepatitis C				
No Yes	REF 5.73 (1.24, 26.51)	0.02	REF 4.54 (0.59, 34.88)	0.15
Pregnancy				
No Yes	REF 1.96 (1.00, 3.84)	0.05	REF 1.69 (0.74, 3.86)	0.21
STIs				
No Yes	NA	NA	NA	NA

**Fig. 1:**
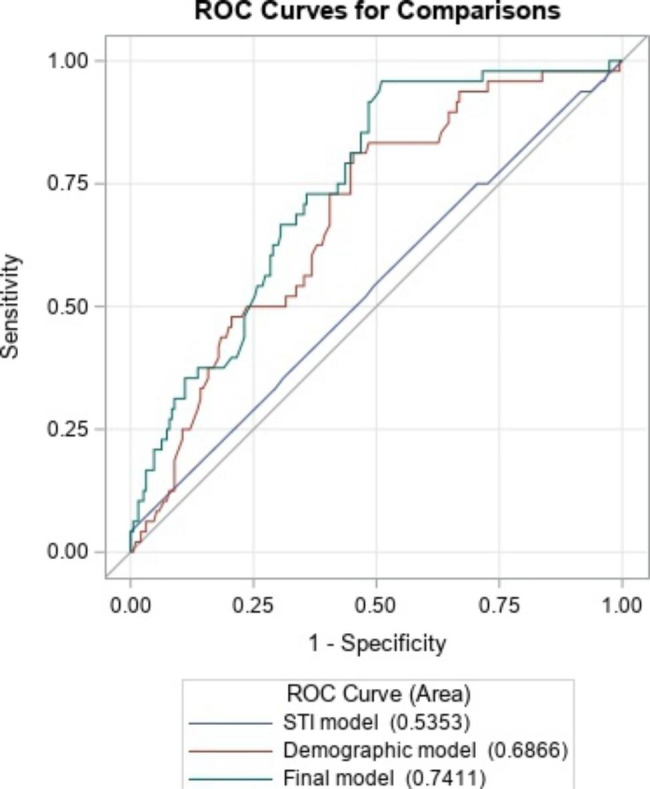
Area under the curve results from various logistic regression models for the outcome of being newly diagnosed with HIV. (STI model: STIs, healthcare site, number of encounters. Demographic model: Race, ethnicity, age group, healthcare site, number of encounters. Final model: STIs, hepatitis C, pregnancy, race, ethnicity, age group, healthcare site, number of encounters)

The final model included the following variables: STIs, substance use, hepatitis C, pregnancy, race, ethnicity, age group, healthcare site and number of encounters, with an AUC of 0.74 95%CI (0.67, 0.81). This final model performs significantly better than a baseline model consisting only of matching factors and STI diagnoses in the past two years (AUC 0.54 95%CI (0.44, 0.63) (ROC contrast test p-value = 0.004)), as well as a model consisting only of matching factors and demographic factors including race, age group, and ethnicity (AUC 0.69 95%CI (0.61, 0.77) (ROC contrast test p-value = 0.03)) (Fig. 1).

## Discussion

In this study we created a sex-specific model that identified factors associated with HIV incidence among women. Our final model had modest performance, with an AUC of 0.74 95%CI (0.67, 0.81), and compared favorably to both the baseline model and a secondary model. Most notably, the final model identified three relatively novel factors - pregnancy in the last two years, hepatitis C diagnosis in the last two years, and diagnosis of substance use in the last two years - that may assist in identifying women at increased risk for HIV using EMR data. This information may also assist individual providers in identifying women who may need targeted and more frequent HIV screening or women who may be good candidates for PrEP.

Our paper is not the first to use data from women to create a specific model for HIV. Tang et al. created three different machine learning models for populations with different risk factors: injection drug users (IDU), MSM, and female sex workers. Tang et al. also found that hepatitis C test results were important in predicting HIV status among female sex workers [13]. Similarly, Burns et al. created two machine learning models to identify factors associated with HIV among women. These models identified historical drug use but not hepatitis C positive viral testing, as positively associated with incident HIV diagnoses [26].

There may be multiple reasons for the identification of recent pregnancy and recent hepatitis C diagnosis as significant, but relatively novel risk factors for HIV acquisition among women. Pregnancy is a high specificity variable that could serve as a proxy for condomless (or condom failure) sex that increases the risk of potential HIV transmission. Late pregnancy and the postpartum period have also been found to be times when HIV transmission per sex act is increased among serodiscordant couples, [27] with the suggestion of a biologic reason for increased risk. It is also possible that pregnancy and early childrearing time periods capture changes in partner behavior that increase HIV risk, such as less frequent condom use and men being more likely to seek other partners during pregnancy, both of which have been reported in data from sub-Saharan Africa [28]. The other identified risk factor, hepatitis C, may be a proxy for IDU, with historical data suggesting that up to 90% of chronic injection drug users (i.e., those who have injected drugs for 10 or more years) are diagnosed with hepatitis C [29, 30]. Hepatitis C diagnosis being linked to IDU is supported by the fact that the majority of hepatitis C cases within our cohort also had EMR diagnosis codes indicating substance use. Additionally, hepatitis C infection may also be a proxy for either anal sex, or rough vaginal unprotected sex, as hepatitis C is transmitted via blood. Previous work conducted in a sample of mostly Black women in Chicago found 16% of participants self-reported anal sex in the last six months, most of which was condomless [31]. Sexual transmission of hepatitis C has been shown to occur among MSM, although documentation of sexual transmission to women is not as robust [32]. Our finding that hepatitis C is a risk factor for new HIV diagnosis may indicate that women in our study had partners who injected drugs [33]. It is also possible that because hepatitis C transmission appears more common among sexual partners with HIV, hepatitis C diagnosis in our model is somewhat collinear with HIV infection itself [34].

The recognition of recent pregnancy, in particular, as a factor associated with HIV acquisition may be helpful for expanding PrEP discussions to more women who are engaging in unprotected sex. This expansion also fits with the new Centers for Disease Control and Prevention (CDC) PrEP guidelines that suggest all sexually active adult and adolescent patients should receive information about PrEP [35]. Public health practitioners should consider pregnant women and women who have recently given birth to be especially vulnerable to HIV infection. HIV testing during first and third trimesters has been recommended by the American College of Obstetricians and Gynecologists (ACOG) for women who are negative after the first test and “known to be at high risk of acquiring HIV infection,” including those “who reside in jurisdictions with elevated HIV incidence.” [36]. This has relevance for our population, as Chicago is within Cook County, a priority area under the Ending the HIV Epidemic (EHE) plan, identifying it as a high incidence area [37]. Based on this ACOG recommendation, it is likely that the women in our study underwent both first and third trimester HIV screening/rapid screening during labor and delivery. Although we do not know what gestational week women in our population received pregnancy diagnosis codes at RUMC or UCM, the fact that so few HIV cases were gained with expansion of the lookback period suggests that the majority of these women were either pregnant or had recently given birth at the time of their HIV diagnosis. Further work should examine if an additional screening for women in the postnatal period is needed to identify those who may still be vulnerable to HIV. Lastly, although there is evidence to suggest that pregnancy is associated with HIV transmission, it is also possible that due to ACOG screening recommendations pregnancy is serving as an indicator of increased HIV testing rather than HIV transmission itself. Regardless, this finding reemphasizes the importance of HIV screening during pregnancy to identify persons who have been newly infected with HIV or who have been undiagnosed until pregnancy screening.

Our model also identified risk factors that have been previously found to be associated with acquisition of HIV. Recent history of a bacterial STI has been used as an eligibility criterion for PrEP by the CDC since 2017, [38] due to plausible social and biologic mechanisms by which bacterial STIs could increase HIV risk. Although our lookback period was longer than that used by the CDC (two years vs. six months), we found that women with a history of STIs were more likely to be eventually diagnosed with HIV. In fact, both the women in our study who were diagnosed with STIs were also eventually diagnosed with HIV. We also found that drug use was associated with being newly diagnosed with HIV, a finding that has been previously reported among urban women at risk for HIV who suffer from the syndemic of drug use, violence and sexual risk behaviors [39, 40]. Although all of these syndemic components are stigmatized and unlikely to be revealed by patients during a visit, drug use is perhaps the most medically observable and the easiest to record in structured EMR fields. It is therefore possible that drug use in our model serves as a marker for one or more of these syndemic components.

Our findings also reinforce previously established racial disparities in incident HIV cases in the United States. In our study, the women newly diagnosed with HIV were more likely to be Black. Additionally, our general patient population was more likely to be from the West or South sides of Chicago, which are areas with increased minority populations. This inequity reinforces that even though the CDC recommends that all sexually active adults receive information about PrEP, some populations have an increased need, including minority women within the city of Chicago.

This study has some limitations. Despite combining HIV screening information from two major urban hospital opt-out screening programs, the number of incident cases among women was relatively small. The small sample size limited the complexity of the models we could create, as well as the number of risk factors that could be entered into models. We only included three STIs (chlamydia, gonorrhea, and syphilis), but there are other STIs like Human papillomavirus (HPV) that may increase risk for HIV transmission [41]. Unfortunately, we did not measure HPV infection in our study population. Inclusion of other STIs into our model would likely have increased the number of people with diagnosed STIs, and may have either strengthened or weakened our finding that STI diagnoses are a risk factor for new HIV diagnoses. Our data was based on women who had an ED or OBGYN visit with HIV screening at two academic medical centers. Our results may not be generalizable to different patient populations (e.g., migrant women) or to women who receive HIV screening in other settings. It is possible that our study identified risk factors that are different from those that would be identified had we included women who use their primary care provider for STI screening or who are tested for HIV in other hospital departments (e.g., inpatient). It is difficult to determine the directionality of this bias on risk factors we did identify. Our data also contained variables that were poorly reported or completely missing from the EMR, particularly variables on condom use, male sexual partners, sexual activity, and education level. This incomplete information made it difficult to assess these factors, although they may have added greatly to the predictive power of the model. Unfortunately, it is likely that the low level of documentation seen in our EMR systems is reflective of larger unwillingness and lack of time among healthcare providers to document this information, especially for women who appear to be at low risk or during visits that are unrelated to sexual health. To increase ability to use these variables in EMR modeling applications, interventions that normalize discussions of PrEP and sexual health should be promoted. Lastly, although we believe that the majority of our sample was composed of cisgender women (women both born female and who currently identify as female), the lack of EMR variables that specify both birth sex and current gender identity prevented us from ensuring our sample contained only ciswomen.

Additional studies to identify persons at high risk for HIV should consider the use of stratified models, as they may be necessary to fully determine the ways in which different people are vulnerable to HIV. Future work to identify risk factors for women should be conducted among a consortium of healthcare centers or a large healthcare network that would permit the creation of a larger cohort of newly positive women. A larger sample size would allow for the use of a machine learning approach, and also support a more thorough examination of all possible EMR risk factors.

## Conclusion

Overall, this study created a model with acceptable discrimination to determine women with new HIV diagnoses (AUC of 0.74 95%CI (0.67, 0.81)). We identified risk factors including pregnancy, hepatitis C diagnosis, substance use diagnosis and STI diagnosis in a two-year period prior to HIV diagnosis that can be used to identify women who are vulnerable to HIV and would benefit from PrEP.

## Electronic supplementary material

Below is the link to the electronic supplementary material.

Additional File Table 1: ICD9/10 codes used to determine disease diagnoses

## Data Availability

The datasets analyzed during the current study are available from the corresponding author on reasonable request.

## Abbreviations

MSM: Men who have sex with men
AUC: Area under the curve
STI: Sexually transmitted infections
EMR: Electronic Medical Record
PrEP: Pre-exposure prophylaxis
OBGYN: Obstetrics and Gynecology
ED: Emergency Department
UCM: University of Chicago Medicine
RUMC: Rush University Medical Center
CDPH: Chicago Department of Public Health
OR: Odds ratios
aOR: Adjusted odds ratios
95%CI: 95% Confidence interval
EHE: Ending the HIV Epidemic
IDU: Injection drug use
CDC: Centers for Disease Control and Prevention
ACOG: American College of Obstetricians and Gynecologists
